# Brunner's Gland Hyperplasias and Hamartomas in Association with* Helicobacter pylori*

**DOI:** 10.1155/2019/6340565

**Published:** 2019-05-02

**Authors:** Sabahattin Destek, Vahit Onur Gul

**Affiliations:** ^1^Department of General Surgery, Bezmiâlem Vakif University Medical Faculty Hospital, 34093 İstanbul, Turkey; ^2^General Surgery Clinic, Gülhane Training and Research Hospital, 06010 Ankara, Turkey

## Abstract

**Background:**

The proliferative lesions of the Brunner's glands (BGs) are hyperplasia and hamartomas, and they are usually asymptomatic and very rarely diagnosed. The aetiology of these lesions is not yet clear. The aim of this study is to evaluate the clinical presentations of patients with BG hyperplasia and hamartomas and to assess the pathological features of these lesions in association with* Helicobacter pylori* (*H. pylori*).

**Methods:**

Our retrospective study included patients who underwent upper gastrointestinal system endoscopy between 2010 and 2015. The hospital records of 18 patients diagnosed with hyperplasia or hamartoma of BG were reviewed for the clinical and pathological findings. Data from patients with BG lesion were compared with 37 patients who had nonspecific duodenitis as the control group.

**Results:**

Female/male ratio in our study sample was 1/1. The age range was between 16 and 85 years with a mean age of 48.61. BG hyperplasia and hamartomas were found in 72.22 and 27.78% of the patients, respectively. The rate of* H. pylori* in gastric mucosa was 43.2% in the control group and 66.7% in the BG lesion group. In the BG lesion group, the rate of* H*.* pylori* was higher.* H. pylori* was identified in 60% of BG hamartomas and in 69.2% of hyperplastic BGs.

**Conclusion:**

Our study demonstrated that* H. pylori* may play an important role in the development of BG hyperplasia and hamartomas in association with chronic gastritis and duodenitis. This is probably due to chronic irritation.

## 1. Introduction

Brunner's glands (BGs) are branched acinotubular glands and they are most common in the duodenal bulb [1]. Cruveilhier was the first to define BG adenomas in 1835 [1, 2]. In 1934, Feyrter classified these lesions as type 1 nodular hyperplasia, type 2 circumscribed hyperplasia, and type 3 glandular adenoma [3]. The size of BG hyperplasia lesions is up to 5 millimeters (mm) according to some sources and up to 10 mm according to other sources. Larger lesions were called BG hamartomas, adenomas, or brunneromas [3–6].

Proliferative lesions of BG account for 5-10% of all benign duodenal masses and less than 1% of all gastrointestinal tumours [1, 3, 6]. They are often identified incidentally during endoscopy [1, 7]. These lesions are considered benign; however, malignant cases have also been reported [1, 3].

The aetiology of these lesions has not yet been completely clarified [1, 8]. The suggested causes of these lesions include hyperchlorhydria and chronic local irritation,* Helicobacter pylori* (*H. pylori*) infection, chronic pancreatitis, and pancreatic exocrine insufficiency [1, 6].

The role of* H. pylori* infection in the pathogenesis of BG proliferative lesions remains unclear. This study examined the characteristics of the proliferative lesions of BG and examined their association with gastritis, duodenitis, and* H. pylori*.

## 2. Methods

### 2.1. Patients

This retrospective study included patients who underwent an upper gastrointestinal system endoscopy in the endoscopy unit of our hospital during 2010–2015. A review of the patient records revealed that 3671 patients underwent an upper gastrointestinal system endoscopy due to dyspepsia, and stomach and duodenum biopsies were performed in 245 patients. Of these patients, a proliferative lesion of BG was identified in 18 patients. The age, gender, and histopathological findings in the biopsy samples of the gastric and duodenal mucosa; any presence of gastritis, duodenitis and* H. pylori* in the gastric mucosa; the type, size (mm), and the site of the proliferative lesion in BG; and the applied treatment methods were analysed [4, 6]. Clinical and pathological features of the patients with BG lesions were compared with 37 patients who had nonspecific duodenitis. Both groups were evaluated statistically.

### 2.2. Endoscopy and Biopsy Sampling

**Figure 1:**
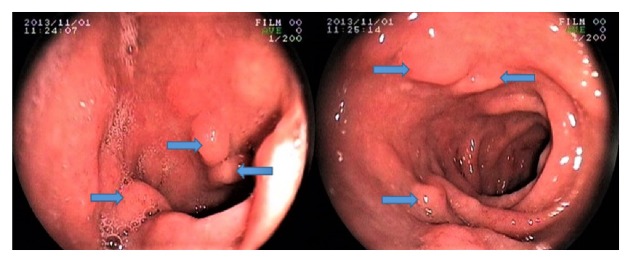
Brunner's gland hamartoma in the duodenal bulb.

Patient reports of the esophago-gastro-duodenoscopies (EGD) performed with Fujinon video endoscopy equipment (Model EG-250WR5, Tokyo, Japan) were reviewed and data were collected. The biopsy results of patients who had biopsies and polypectomies of lesions of the stomach and duodenal mucosa were checked (Figure 1).

### 2.3. Histopathological Examination

Biopsy samples were fixed in 10% formalin for histological examination and then embedded in paraffin blocks. Serial sections of 4 *μ*m (microns) were prepared from these blocks. After deparaffinization, the tissue sections were stained with haematoxylin and eosin and Giemsa for histological examination and for identifying* H. pylori*, respectively. The slides were examined by a pathologist experienced in light microscopy.

**Figure 2:**
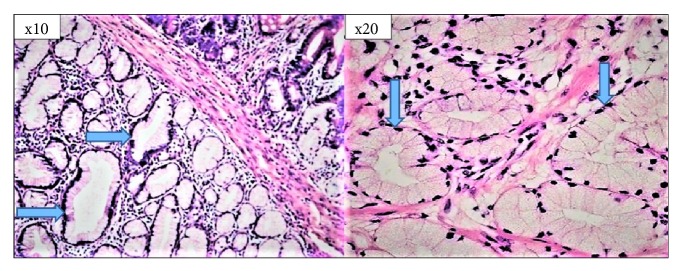
Brunner's gland hamartoma in the duodenal bulb (H–E; ×10 and ×20).

Gastric and duodenal biopsy specimens were evaluated according to the updated Sydney system [9]. In the histological examination, the presence or absence of* H. pylori* was recorded as “yes” or “no”, respectively, and the grading was recorded as “no* H. pylori*” or “mild”, “moderate”, or “severe* H. pylori ***”** based on the updated Sydney system [10]. BG lesions were classified according to their size. Lesions with hamartoma less than 5 mm and lesions less than 5 mm were considered as hyperplasia [2–4] (Figure 2).

### 2.4. Statistical Analysis

The study data were analysed using licenced SPSS 21 package software (IBM Corp., Armonk, NY, USA). The Mann-Whitney U test was used to test the differences between the groups if data did not conform to a normal distribution. The Chi-square test was used to analyse the associations of the groups of nominal variables. If the nominal values were not sufficient in the cells of 2 × 2 tables, Fisher's exact test was used. The Pearson Chi-square (X^2^) test with Monte Carlo simulation was used to analyse r x c (row, column) tables. The results were interpreted at a significance level of 0.05. A level of* p* < 0.05 indicated a significant difference, whereas* p* > 0.05 indicated that there was no significant difference.

## 3. Results

The female/male ratio of BG lesion patients included in the study was 1:1. The mean age of the patients was 48.6 years. The patients had chronic gastritis and chronic duodenitis at rates of 61.11 and 88.89%, respectively. The examinations revealed that 66.7% of the BG lesion patients had* H. pylori* in the gastric mucosa. The control group was nonspecific duodenitis patients with a female/male ratio of 2.4 and the mean age of the patients in this group was 39.4 years. In 43.2% of nonspecific duodenitis patients,* H. pylori* was found in the gastric mucosa. Patients with BG lesion had more* H. pylori* in gastric mucosa than patients with nonspecific duodenitis (Table 1).

BG hyperplasia and hamartomas were found in 72.22 and 27.78% of the patients, respectively. The sections with BG hyperplasia had a diameter of 2–4 mm (with a mean diameter of 3 mm). BG hamartomas had a diameter between 5–30 mm (with a mean of 12.8 mm). Polypectomy was performed for BG hamartomas larger than 10 mm in order to eliminate the risk of bleeding and malignancy. Polypectomy was performed in three of the patients. No malignancies were diagnosed in the study patients. Yearly follow-ups with endoscopic examination were scheduled for the patients (Table 2).

In our study, 61.54% of the cases of BG hyperplasia occurred in males; 80% of BG hamartomas were seen in women. However, no significant differences in gender were detected. In our study, there were statistically significant differences in the type and localization of the proliferative lesion of BG (*p* = 0.046). While BG hamartomas were most commonly found posteriorly, BG hyperplasia occurred in both aspects of the duodenum. A statistically significant difference was found between the type of the proliferative lesion and the age of the patients (*p* = 0.002). BG hamartomas are seen in advanced age. A statistically significant difference was found between the diameter and type of proliferative lesion (*p* = 0.0001). The mean lesion diameter of BG hamartomas was statistically significantly higher. However, there was not a statistically significant difference between the type of the proliferative lesion of BG and the other variables (*p* > 0.05) (Table 2).

**Figure 3:**
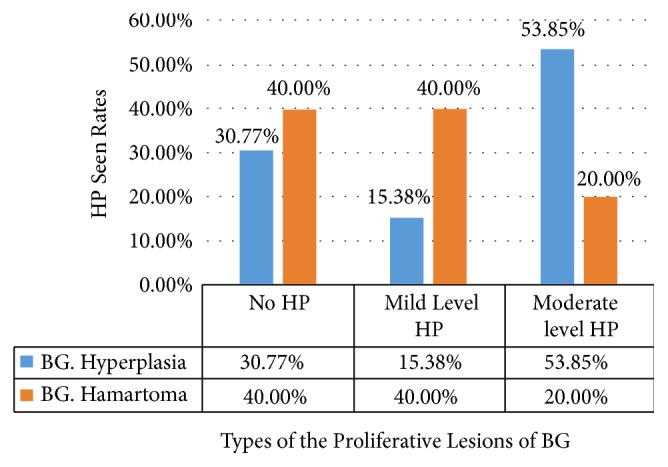
Presence of* Helicobacter pylori* (HP) in the proliferative lesions of Brunner's gland (BG).

Chronic gastritis and chronic duodenitis were found in 61.54 and 84.62% of patients, respectively. Chronic gastritis was present in 60% of the patients with BG hamartoma. Chronic duodenitis was found in all study patients. After duodenal biopsy, there was* H. pylori* in the gastric mucosa, and 66.7% of those had BG lesions; 49.63% of patients had non-BG lesions, such as nonspecific duodenitis and enteropathy.* H. pylori* was found in 69.23% of hyperplastic BGs.* H. pylori* density was mild or moderate in 22.2% and 77.8% of the patients with BG hyperplasia.* H. pylori* was detected in the stomach mucosa of 60% of patients with a BG hamartoma.* H. pylori* density was mild in 66.7% and moderate in 33.3% of these patients (Figure 3).

No recurrences were found during the scheduled yearly follow-ups in the patients who underwent a polypectomy. Eradication treatment was performed in patients with* H. pylori*. No pathological changes or signs of malignancy were found in the yearly routine endoscopic follow-ups in patients who were given treatment and underwent a diet for dyspepsia.

## 4. Discussion

Most BGs are located proximal to the ampulla of Vater, decreasing in number towards the distal duodenum [1, 2]. BGs in submucosa contain cells secreting zymogens and mucus [10]. These glands allow for the neutralization of chyme (i.e., the mixture of food and acid coming from the stomach) by secreting approximately 200 milliliters of an alkaline mucus (with a pH between 8.1–9.3) daily [5, 11]. BGs secrete pepsinogen, urogastrone, and enterokinase [11].

Feyrter classified the abnormal proliferation of BGs in 1934. According to this classification, multiple areas of sessile nodular hyperplasia along the duodenum are named as type 1, sessile nodular hyperplasia in the duodenal bulbus are named as type 2, and polypoid tumour-like glandular adenomas are named as type 3 [1, 3, 12]. The most common form is type 2 [3].

BG hyperplasia consists of many small polypoid lesions of excessive BG separated by fibrous septa. A BG hamartoma consists of a single mass that contains a mixture of acini, channels, smooth muscle, adipose tissue and lymphoid tissue [4, 6]. Pathologists believe that these lesions are caused by the same pathological process [4, 11, 12]. BG hamartomas today are not called BG adenomas because they do not show cellular atypia [3, 4]. Researchers classify lesions smaller than either 5 or 10 mm as BG hyperplasia and larger lesions as hamartomas [4, 12, 13]. We applied this classification to our study.

In various patients with routine EGDs, BG hyperplasia was found in 0.3% of the sample [14]. In EGD series, 2.2–7.6% of duodenal biopsies were diagnosed with BG hyperplasia [7, 12–14]. They usually occur as multiple small nodular lesions in the first part of the duodenum [3], with a cobblestone appearance on the mucosa [3, 6]. Hyperplastic BGs generally display an equal gender distribution and are seen at around 50 years of age [1, 6]. However, some studies report a higher incidence in men [12]. The major complaint of the patients with hyperplastic BGs is dyspepsia [3, 6].

BG hamartomas are found in 0.01–0.07% of routine EGD patients [14–16]. They constitute 5–10% of benign duodenal masses [17–20]. They are generally seen in the posterior wall of the first part of the duodenum and are solitary (57–70%) [4, 8, 20, 21]. They may vary in size between 0.5 and 12 cm. They appear in the shape of polyps with a stalk in 88 to 89% of the cases, and 11 to 12% of them appear as sessile polyps [8, 21]. The age range is 15–80, with a usual occurrence in the fifth decade [4, 8, 21]. In some studies, they were found to be more common in men and in others they were found to be more common in women [4, 8, 21]. Of the symptomatic patients, 28–61% may present with gastrointestinal haemorrhage, 51% with abdominal pain, and 44–50% with nausea and vomiting. Rarely there are signs of intestinal obstruction, duodenal intussusception, obstructive jaundice, or recurrent pancreatitis [1, 20, 21].

In our study, BG hyperplasia was found in 0.35% of all patients who underwent EGDs and in 5.3% of the patients who underwent duodenal biopsies. The mean age of the patients was 40.9 years. The female/male ratio was 0.6. The mean diameter of the lesions was 3 mm, and they were mostly located in the bulb. BG hamartomas were found in 0.13% of the patients who underwent EGD and in 2% of the patients from whom duodenal biopsy specimens were collected. The mean age of the patients was 68.6 years. The female/male ratio was 4. The mean diameter of the lesions was 12.8 mm, and the lesions were most commonly located at the posterior duodenum. All patients presented with nonspecific complaints.

Diagnosis is usually confirmed by imaging studies and EGD [1]. Endoscopy, abdominal tomography, endoscopic sonography, barium X-rays, fluoroscopic examination, and transabdominal sonography are used as diagnostic tests [20–22]. Proliferative lesions of BG are usually considered as benign [12, 14]. However, some studies reported epithelial dysplasia or adenocarcinomas [1, 13, 23]. In our study, dysplasia and malignancy were not detected in the lesions.

Differential diagnosis for these lesions includes adenomatous polyps, lipomas, leiomyomas, lymphomas, ectopic pancreatic tissue, gastrointestinal tumours, etc. [21, 22]. In proliferative lesions of BG, conservative treatments may be administered, accompanied with regular follow-ups using endoscopy [20, 21]. The endoscopic removal of BG hamartomas should be considered to establish a definitive diagnosis and to prevent potential complications, including bleeding or obstruction [21, 22]. Surgery is recommended in patients with wider pedunculated or sessile BG hamartomas, in patients with unsuccessful endoscopic interventions and in patients in whom a malignancy is suspected [24, 25]. In our study, no surgical intervention was needed.

The pathogenesis of proliferative lesions of BGs has not yet been completely elucidated [1]. The main aetiology is suggested to be a chronic local irritation in the duodenal mucosa [26]. The major factors studied in mucosal injury are gastric hyperacidity, mechanical stimuli in the duodenum and* H. pylori* infections [26, 27]. It has been reported that BG hyperplasia especially develops in patients with chronic gastritis and duodenal ulcers in the presence of hyperchlorhydria [13, 20]. BG hyperplasia has been reported to present comorbidly with peptic ulcers, chronic renal failure and chronic pancreatitis [20, 26, 28]. However, this opinion has not been widely accepted either.

*H. pylori* is the major cause of several gastroduodenal diseases, including chronic gastritis, duodenitis and duodenal ulcers [29]. Studies have found that gastric non-Hodgkin lymphoma formation was involved in the etiology of* H. pylori,* especially in the formation of esophageal and gastric cancer [30, 31].* H. pylori* eradication therapy was found to decrease the risk of malignancy [30, 31]. However, the role of* H. pylori* in the etiology of rare duodenal malignancies remains unclear [31]. It has been suggested that* H. pylori* infection may be involved in the pathogenesis of BG hyperplasia [1, 32]. Studies have reported that* H. pylori* was detected in 71% of the patients with BG hamartomas and that* H. pylori* may induce proliferative processes in BGs. It has also been reported that* H. pylori* infections and chronic gastritis may induce the progression of a hyperplastic lesion to a hamartoma [33, 34]. There are also other reports that do not address* H. pylori* in the aetiology of these lesions or state that it was not associated with these type of lesions [4, 12, 20].

In our study, 61.11% of the patients had chronic gastritis, 88.89% had chronic duodenitis and 66.7% had* H. pylori* in the gastric mucosa.* H. pylori* was detected in BG hamartomas and hyperplastic BGs in our study at rates of 60 and 69.2%, respectively. This suggests that* H. pylori* may play an important role in the aetiology of BG proliferative lesions due to the chronic local irritation associated with both chronic gastritis and chronic duodenitis.

## 5. Conclusions

BG hyperplasia and hamartomas are usually rare benign duodenal lesions that are detected incidentally. The pathogenesis of these lesions is still unclear. Several investigators have suggested a variety of causal factors. In our study, we found that BG hyperplasia and hamartomas were associated with a high rate of chronic gastritis, chronic duodenitis, and the presence of* H. pylori*. We are of the opinion that* H. pylori* should be considered in the pathogenesis and treatment of BG hyperplasia and hamartomas.

## Figures and Tables

**Table 1:** Distribution of clinical and pathological features.

Clinical and pathological features		Control Group		BG ^a^ Lesion Group		Statistical result
		(Nonspecific duodenitis)				
		n	%	n	%	*p*

Gender	Female	26	70.3	9	50	0,208 ^b^
	Male	11	29.7	9	50	
Gastritis Status	Chronic gastritis	19	51.4	11	61.1	0,49 ^b^
	Active chronic gastritis	18	48.6	7	38.9	
Duodenitis Status	Chronic duodenitis	37	100	16	88.9	0.103 ^c^
	Active chronic duodenitis	0	0	2	11.1	
*H. pylori* ^f^ Status	No	21	56.8	6	33.3	0,103 ^b^
	Yes	16	43.2	12	66.7	

Variable		n Mean Min.		Max.	SD ^e^	

Age	Control Group	37 39.4 14		71	15,3	0,086 ^d^
	BG Lesion Group	18 48.6 16		85	18,3	

^a^BG = Brunner's gland, ^b^ X^2^ = Chi-square test, ^c^ f = Fisher's exact test, ^d^ U = Mann-Whitney U test, ^e^ SD = standard deviation, and ^f^ *H. pylori *= *Helicobacter pylori*.

**Table 2:** Association between the proliferative lesions of Brunner's gland and the variables.

Variables		Type of the Proliferative Lesion of BG ^a^				Statistical result
		Hamartoma		Hyperplasia		
		*n*	%	*n*	%	*p* ^b^

Gender	Female	4	80	5	38.46	0.294
	Male	1	20	8	61.54	
Gastritis Status	Chronic gastritis	3	60	8	61.54	0.676
	Active chronic gastritis	2	40	5	38.46	
Lymphoid Hyperplasia	No	3	60	11	84.62	0.533
	Yes	2	40	2	15.38	
Foveolar Hyperplasia	No	5	100	12	92.31	0.722
	Yes	0	0	1	7.69	
Gastric Mucosa Inflammation	No	0	0	0	0	-
	Yes	5	100	13	100	
Gastric Mucosa Activation	No	3	60	6	46.15	0.5
	Yes	2	40	7	53.85	
Gastric Mucosa Metaplasia	No	4	80	13	100	0.278
	Yes	1	20	0	0	
Gastric Mucosa Atrophy	No	4	80	13	100	0.278
	Yes	1	20	0	0	
Gastric Mucosa *H. pylori* ^d^	No	2	40	4	30.77	0.561
	Yes	3	60	9	69.23	
Duodenitis Status	Chronic duodenitis	5	100	11	84.62	0.51
	Active chronic duodenitis	0	0	2	15.38	
Site of the Proliferative Lesion of BG	Anterior bulbus	4	80	3	23.08	**0.046**
	Posterior bulbus	1	20	2	15.38	
	On both sides	0	0	8	61.54	

Variables		Type of the Proliferative Lesion of BG				
		*n*	Mean Min. Max.		SD ^e^	*p* ^c^

Age	BG hamartoma	5	68.6 52 85		11.93	**0.002**
	BG hyperplasia	13	40.92 16 63		13.97	
Lesion diameter (mm)	BG hamartoma	5	12.8 5 30		9.88	**0.0001**
	BG hyperplasia	13	3.07 2 4		0.8	

^a^ BG = Brunner's gland, ^b^ f = Fisher's exact test, ^c^ U = Mann-Whitney U test, ^d^ *H. pylori* = *Helicobacter pylori*, and ^e^ SD = standard deviation.

## Data Availability

The data used to support the findings of this study have been deposited in the Harvard Dataverse repository [https://doi.org/10.7910/DVN/ZCLGFV] (https://www.re3data.org/repository/r3d100010051).

## References

[1] Abbass R., Al-Kawas F. H. (2008). Brunner gland hamartoma. *Gastroenterology & Hepatology*.

[2] Gao Y.-P., Zhu J.-S., Zheng W.-J. (2004). Brunner's gland adenoma of duodenum: a case report and literature review. *World Journal of Gastroenterology*.

[3] Feyrter F. (1938). Uber wucherunger der Brunnerschen Drusen. *Virchows Archiv : An İnternational Journal of Pathology*.

[4] Patel N. D., Levy A. D., Mehrotra A. K., Sobin L. H. (2006). Brunner's gland hyperplasia and hamartoma: imaging features with clinicopathologic correlation. *American Journal of Roentgenology*.

[5] Maglinte D. D., Mayes S. L., Ng A. C., Pickett R. D. (1982). Brunner's gland adenoma: diagnostic considerations. *Journal of Clinical Gastroenterology*.

[6] Rocco A., Borriello P., Compare D. (2006). Large Brunner's gland adenoma: case report and literature review. *World Journal of Gastroenterology*.

[7] Terada T. (2012). Pathologic observations of the duodenum of 615 consecutive duodenal specimens: 1. benign lesions. *International Journal of Clinical and Experimental Pathology*.

[8] Levine J. A., Burgart L. J., Batts K. P., Wang K. K. (1995). Brunner's gland hamartomas: clinical presentation and pathological features of 27 cases. *American Journal of Gastroenterology*.

[9] Sipponen P., Price A. B. (2011). The Sydney system for classification of gastritis 20 years ago. *Gastroenterology and Hepatology*.

[10] Manxhuka-Kerliu S., Telaku S., Devolli-Disha E. (2009). Helicobacter pylori gastritis updated Sydney classification applied in our material. *Prilozi / Makedonska Akademija Na naukite i Umetnostite, Oddelenie Za Biološki i Medicinski Nauki*.

[11] Collaco A. M., Jakab R. L., Hoekstra N. E., Mitchell K. A., Brooks A., Ameen N. A. (2013). Regulated traffic of anion transporters in mammalian Brunner's glands: a role for water and fluid transport. *American Journal of Physiology-Gastrointestinal and Liver Physiology*.

[12] Kim K., Jang S. J., Song H. J., Yu E. (2013). Clinicopathologic characteristics and mucin expression in Brunner's gland proliferating lesions. *Digestive Diseases and Sciences*.

[13] Franzin G., Musola R., Ghidini O., Manfrini C., Fratton A. (1985). Nodular hyperplasia of Brunner's glands. *Gastrointestinal Endoscopy*.

[14] Jung S. H., Chung W. C., Kim E. J. (2010). Evaluation of non-ampullary duodenal polyps: Comparison of non-neoplastic and neoplastic lesions. *World Journal of Gastroenterology*.

[15] Sedano J., Swamy R., Jain K., Gupta S. (2015). Brunner's gland hamartoma of the duodenum. *Gastrointestinal Endoscopy*.

[16] Botsford T. W., Crowe P., Crocker D. W. (1962). Tumors of the small intestine. A review of experience with 115 cases including a report of a rare case of malignant hemangio-endothelioma. *The American Journal of Surgery*.

[17] Culver E. L., McIntyre A. S. (2011). Sporadic duodenal polyps: Classification, investigation, and management. *Endoscopy*.

[18] Dhouha B., Ahlem L., Sana B. S., Saadia B., Sabeh M. R. (2017). Unexpected cause for duodenal obstruction: Brunner's gland hyperplasia. *Pathologica*.

[19] Robertson H. E. (1941). Pathology of Brunner's glands. *Archives of Pathology*.

[20] Houwers J. B., de Bie S. H., Hofstee N. (2012). AIRP best cases in radiologic-pathologic correlation: Brunner Gland Hamartoma. *RadioGraphics*.

[21] Walden D. T., Marcon N. E. (1998). Endoscopic injection and polypectomy for bleeding Brunner's gland hamartoma: case report and expanded literature review. *Gastrointestinal Endoscopy*.

[22] Sakurai T., Sakashita H., Honjo G., Kasyu I., Manabe T. (2005). Gastric foveolar metaplasia with dysplastic changes in Brunner gland hyperplasia: possible precursor lesions for Brunner gland adenocarcinoma. *The American Journal of Surgical Pathology*.

[23] Gaspar J. P., Stelow E. B., Wang A. Y. (2016). Approach to the endoscopic resection of duodenal lesions. *World Journal of Gastroenterology*.

[24] de Nes L., Ouwehand F., Peters S., Boom M. (2008). A large Brunner’s gland hamartoma causing gastrointestinal bleeding and obstruction. *Digestive Surgery*.

[25] Peloso A., Viganò J., Vanoli A. (2017). Saving from unnecessary pancreaticoduodenectomy. Brunner's gland hamartoma: Case report on a rare duodenal lesion and exhaustive literature review. *Annals of Medicine and Surgery*.

[26] Akaki M., Taniguchi S., Hatakeyama K., Kushima R., Kataoka H. (2014). Duodenal mucosal damage is associated with proliferative activity of Brunner's gland hamartoma: a case report. *BMC Gastroenterology*.

[27] Stolte M., Schwabe H., Prestele H. (1981). Relationship between diseases of the pancreas and hyperplasia of Brunner's glands. *Virchows Archiv A: Pathological Anatomy and Histology*.

[28] Chattopadhyay P., Kundu A. K., Bhattacharyya S., Bandyopadhyay A. (2008). Diffuse nodular hyperplasia of Brunner's gland presenting as upper gastrointestinal haemorrhage. *Singapore Medical Journal*.

[29] Sugano K., Tack J., Kuipers E. J. (2015). Kyoto global consensus report on *Helicobacter pylori* gastritis. *Gut*.

[30] Pimentel-Nunes P., Libânio D., Marcos-Pinto R. (2019). Management of epithelial precancerous conditions and lesions in the stomach (MAPS II): European Society of Gastrointestinal Endoscopy (ESGE), European Helicobacter and Microbiota Study Group (EHMSG), European Society of Pathology (ESP), and Sociedade Portuguesa de Endoscopia Digestiva (SPED) guideline update 2019. *Endoscopy*.

[31] Lam S., Yu J., Wong S., Peppelenbosch M., Fuhler G. (2017). The gastrointestinal microbiota and its role in oncogenesis. *Best Practice & Research Clinical Gastroenterology*.

[32] Peetz M. E., Moseley H. S. (1989). Brunner's gland hyperplasia. *The American Surgeon*.

[33] Kovacevi I., Ljubici N., Cupi H. (2001). *Helicobacter pylori *infection in patients with Brunner’s gland adenoma. *Acta Medica Croatica*.

[34] Kurella R. R., Ancha H. R., Hussain S., Lightfoot S. A., Harty R. (2008). Evolution of brunner gland hamartoma associated with helicobacter pylori infection. *Southern Medical Journal*.

